# Polo-like kinase 1 inhibition sensitizes neuroblastoma cells for vinca alkaloid-induced apoptosis

**DOI:** 10.18632/oncotarget.3901

**Published:** 2015-05-14

**Authors:** Sebastian Czaplinski, Manuela Hugle, Valerie Stiehl, Simone Fulda

**Affiliations:** ^1^ Institute for Experimental Cancer Research in Pediatrics, Goethe-University, Frankfurt, Germany; ^2^ German Cancer Consortium (DKTK), Heidelberg, Germany; ^3^ German Cancer Research Center (DKFZ), Heidelberg, Germany

**Keywords:** PLK1, apoptosis, BCL-2, mitosis, neuroblastoma

## Abstract

High polo-like kinase 1 (PLK1) expression has been linked to poor outcome in neuroblastoma (NB), indicating that it represents a relevant therapeutic target in this malignancy. Here, we identify a synergistic induction of apoptosis by the PLK1 inhibitor BI 2536 and vinca alkaloids in NB cells. Synergistic drug interaction of BI 2536 together with vincristine (VCR), vinblastine (VBL) or vinorelbine (VNR) is confirmed by calculation of combination index (CI). Also, BI 2536 and VCR act in concert to reduce long-term clonogenic survival. Importantly, BI 2536 significantly enhances the antitumor activity of VCR in an *in vivo* model of NB. Mechanistically, BI 2536/VCR co-treatment triggers prolonged mitotic arrest, which is necessary for BI 2536/VCR-mediated apoptosis, since pharmacological inhibition of mitotic arrest by the CDK1 inhibitor RO-3306 significantly reduces cell death. Prolonged mitotic arrest leads to phosphorylation-mediated inactivation of BCL-2 and BCL-X_L_ as well as downregulation of MCL-1, since inhibition of mitotic arrest by RO-3306 also prevents phosphorylation of BCL-2 and BCL-X_L_ and MCL-1 downregulation. This inactivation of antiapoptotic BCL-2 proteins promotes activation of BAX and BAK, cleavage of caspase-9 and -3 and caspase-dependent apoptosis. Engagement of the mitochondrial pathway of apoptosis is critically required for BI 2536/VCR-induced apoptosis, since ectopic expression of a non-degradable MCL-1 phospho-mutant, BCL-2 overexpression or BAK knockdown significantly reduce BI 2536/VCR-mediated apoptosis. Thus, PLK1 inhibitors may open new perspectives for chemosensitization of NB.

## INTRODUCTION

The prognosis of NB, a common solid tumor of childhood, remains poor especially for advanced and relapsed disease [1]. This highlights the demand for the development of novel therapeutic strategies. Since the efficacy of many anticancer therapies including chemotherapy depends on intact cell death programs in cancer cells, reactivation of programmed cell death represents a promising avenue [2].

Apoptosis is a form of programmed cell death that is activated upon treatment with various anticancer drugs [2]. Signaling via the intrinsic (mitochondrial) pathway is critical for proper activation of caspases in the course of anticancer drug treatment and is tightly controlled by pro- and antiapoptotic proteins [3]. For example, antiapoptotic proteins of the BCL-2 family such as BCL-2, BCL-X_L_ and MCL-1 as well as the proapoptotic members BAX and BAK play an important role in regulating mitochondrial outer membrane permeabilization [4].

Induction of programmed cell death by apoptosis is linked to the control of cell cycle progression in order to eliminate cells that exhibit abnormalities during mitosis. Antimitotic drugs have been shown to engage mitotic cell death, although the underlying molecular mechanisms have remained largely elusive [5]. For example, vinca alkaloids that destabilize microtubule formation such as VCR, VBL and VNR are commonly used as anticancer drugs in various cancers including pediatric malignancies [6].

PLK1 is a serine/threonine kinase that plays a critical role in the control of cell cycle progression and mitosis [7]. PLK1 overexpression has been documented in various cancers and has been implied in their malignant phenotype [7]. In NB, elevated expression of PLK1 has recently been associated with high-risk disease and unfavorable patient outcome [8], suggesting that PLK1 represents a relevant therapeutic target in NB.

Several small-molecule PLK1 inhibitors have been developed as cancer therapeutics in recent years and are under investigation in clinical trials [9–11]. PLK1 inhibition has been reported to cause mitotic arrest and initiate apoptosis in various human cancer cell lines [12, 13]. Initial testing of the PLK1 inhibitor BI 6727 by the Pediatric Preclinical Testing Program recently showed that BI 6727 as single agent caused regressions in only a minority of childhood cancer xenograft models including NB [14]. This underlines that rational combination strategies are required to augment the antitumor activity of PLK1 inhibitors. Searching for new and more effective treatment options, we investigated the potential of PLK1 inhibition for chemosensitization of NB.

## RESULTS

### PLK1 inhibitor synergizes with vinca alkaloids to induce apoptosis in NB cells

**Figure 1:**
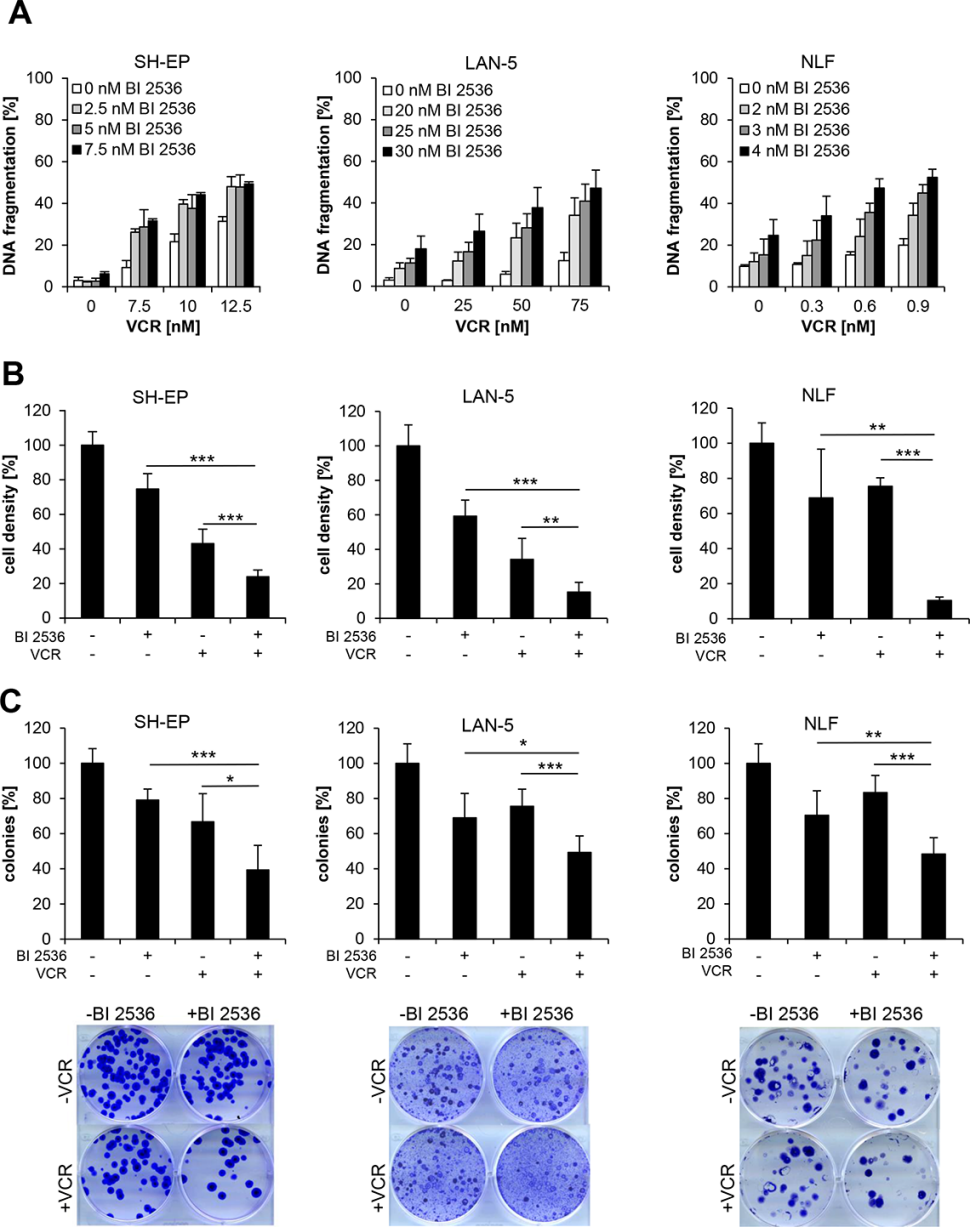
BI 2536 synergizes with vinca alkaloids to induce apoptosis in NB cells **A.** SH-EP, LAN-5 and NLF cells were treated for 48 hours with indicated concentrations of BI 2536 and VCR. Apoptosis was determined by analysis of DNA fragmentation of PI-stained nuclei using flow cytometry. Data are shown as mean +/− SD of three independent experiments performed in triplicate. **B.** SH-EP cells were treated with 7.5 nM BI 2536 and/or 5 nM VCR, LAN-5 cells with 30 nM BI 2536 and/or 75 nM VCR, NLF cells with 4 nM BI 2536 and/or 0.5 nM VCR for 48 hours. Cell density was measured by crystal violet staining and is expressed as a percentage of untreated cells. Data are shown as mean +/− SD of three independent experiments performed in triplicate; **, *P* < 0.01; ***, *P* < 0.001. **C.** SH-EP cells were treated with 7.5 nM BI 2536 and/or 5 nM VCR, LAN-5 cells with 30 nM BI 2536 and/or 30 nM VCR, NLF cells with 4 nM BI 2536 and/or 0.5 nM VCR for 12 hours and subsequently cultured in drug-free medium for additional 13 days. Colony formation was assessed by crystal violet staining and colonies were counted macroscopically. The number of colonies is expressed as percentage of untreated controls (upper panels) and representative images are shown (lower panels). Data are shown as mean +/− SD of three independent experiments performed in triplicate; *, *P* < 0.05; **, *P* < 0.01; ***, *P* < 0.001.

To explore whether PLK1 inhibitors can be exploited to enhance chemosensitivity of NB, we tested the small-molecule PLK1 inhibitor BI 2536 together with subtoxic doses of VCR that is frequently used in standard therapy regimens for the treatment of NB [1]. We found that BI 2536 synergized with VCR to induce apoptosis in NB cells (Figure 1A). Similarly, BI 2536 acted together with other vinca alkaloids such as VBL and VNR to significantly increase DNA fragmentation, used as a characteristic parameter of apoptotic cell death (Supplementary Figure 1). Synergistic drug interaction of BI 2536 together with VCR, VBL or VNR was confirmed by calculation of CI (Supplementary Table 1). Furthermore, we confirmed that BI 2536 and VCR acted in concert to significantly reduce cell viability compared to either agent alone by using crystal violet assay as an additional cytotoxicity test (Figure 1B). Besides these short-term assays, we tested the effects of the drug combination on long-term clonogenic survival by performing colony assays. BI 2536/VCR co-treatment cooperated to significantly reduce colony formation compared to treatment with BI 2536 or VCR alone (Figure 1C). This set of experiments shows that the PLK1 inhibitor BI 2536 synergistically cooperates with vinca alkaloids to induce apoptosis and to reduce long-term clonogenic growth of NB cells.

### BI 2536 and VCR cooperate to activate caspases and caspase-dependent apoptosis

**Figure 2:**
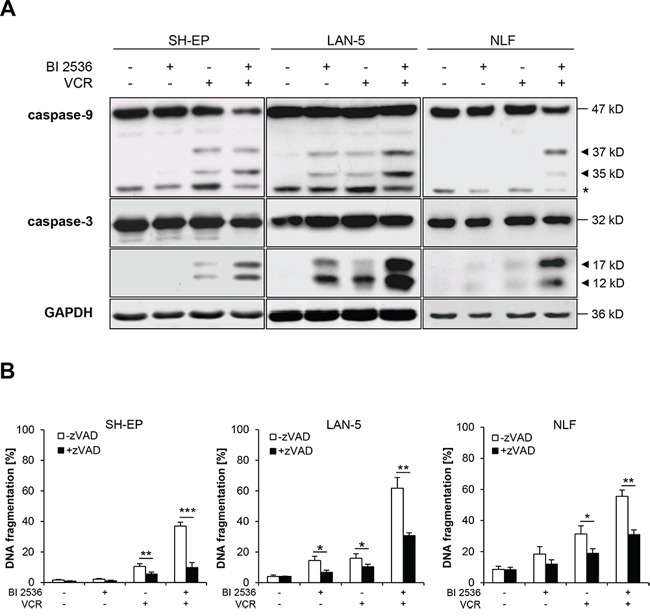
BI 2536 and VCR cooperate to induce caspase-dependent apoptosis **A.** SH-EP cells were treated with 7.5 nM BI 2536 and/or 5 nM VCR, LAN-5 cells with 30 nM BI 2536 and/or 30 nM VCR, NLF cells with 4 nM BI 2536 and/or 0.5 nM VCR for 24 hours. Activation of caspase-9 and -3 was analyzed by Western blotting. Arrowheads indicate active cleavage fragments, expression of GAPDH served as loading control, asterisk denotes unspecified bands. Representative blots of two independent experiments are shown. **B.** SH-EP, LAN-5 and NLF cells were treated for 48 hours as described in (A) in the presence or absence of 20–50 μM zVAD.fmk. Apoptosis was determined by analysis of DNA fragmentation of PI-stained nuclei using flow cytometry. Data are shown as mean +/− SD of three independent experiments performed in triplicate; *, *P* < 0.05; **, *P* < 0.01; ***, *P* < 0.001.

To gain insights into the molecular mechanisms of the synergism of BI 2536/VCR co-treatment, we examined activation of caspases known as key effector molecules of apoptosis by Western blot analysis [15]. BI 2536 and VCR cooperated to induce cleavage of caspase-9 into active p37/p35 fragments and cleavage of caspase-3 into active p17/p12 fragments (Figure 2A). To test whether caspase activity is required for the induction of apoptosis, we used the broad-range caspase inhibitor zVAD.fmk. Addition of zVAD.fmk significantly reduced BI 2536/VCR-induced apoptosis (Figure 2B), demonstrating that caspases are required for the induction of apoptosis upon combined treatment with BI 2536 and VCR.

### Mitotic arrest upon BI 2536/VCR treatment is necessary for apoptosis

**Figure 3:**
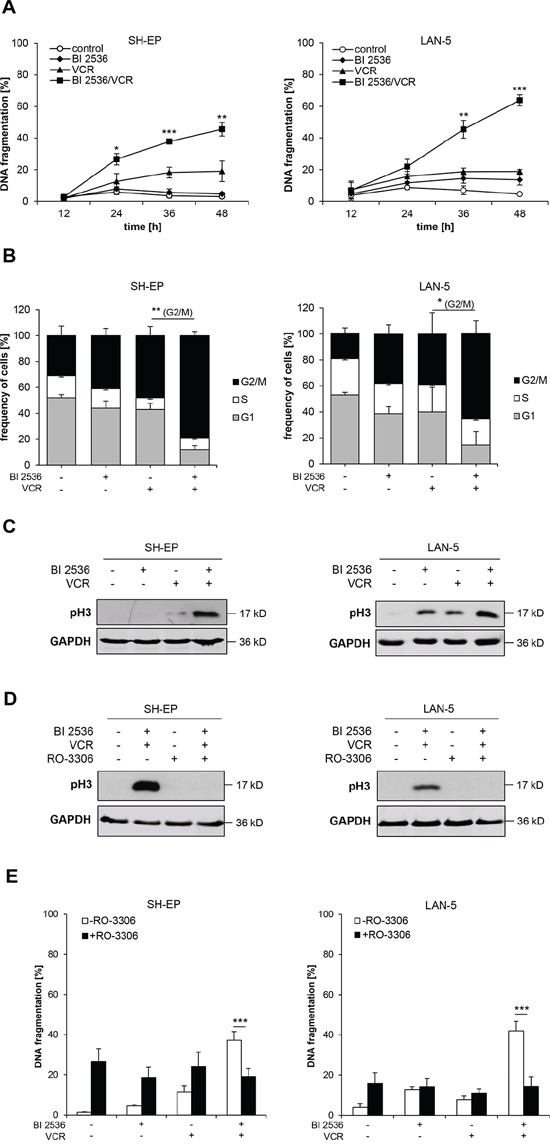
Mitotic arrest is required for BI 2536/VCR-induced apoptosis **A.** SH-EP cells were treated with 7.5 nM BI 2536 and/or 10 nM VCR, LAN-5 cells with 30 nM BI 2536 and/or 75 nM VCR for indicated times. Apoptosis was determined by analysis of DNA fragmentation of PI-stained nuclei using flow cytometry. Data are shown as mean +/− SD of three independent experiments performed in triplicate; *, *P* < 0.05; **, *P* < 0.01; ***, *P* < 0.001 comparing BI 2536/VCR-cotreated to VCR-treated cells. **B.** SH-EP and LAN-5 cells were treated for 12 hours with BI 2536 and/or VCR as described in (A). DNA was stained with PI and cell cycle analysis was performed using FlowJo software. Data are shown as mean +/− SD of three independent experiments performed in triplicate; *, *P* < 0.05; **, *P* < 0.01 comparing BI 2536/VCR-cotreated to VCR-treated cells in G2/M phase. **C.** SH-EP cells were treated with 7.5 nM BI 2536 and/or 5 nM VCR, LAN-5 cells with 30 nM BI 2536 and/or 30 nM VCR for 12 hours, protein lysates were prepared and expression of mitotic marker pH3 was analyzed by Western blot. Expression of GAPDH served as loading control. Representative blots of two independent experiments are shown. **D.** SH-EP and LAN-5 cells were treated for 12 hours with 10 μM RO-3306 and BI 2536 and/or VCR as described in (C), protein lysates were prepared and expression of mitotic marker pH3 was analyzed by Western blot. Expression of GAPDH served as loading control. Representative blots of two independent experiments are shown. **E.** SH-EP and LAN-5 cells were co-treated for 48 hours with 10 μM RO-3306 and BI 2536 and/or VCR as described in (C). Apoptosis was determined by analysis of DNA fragmentation of PI-stained nuclei using flow cytometry. Data are shown as mean +/− SD of three independent experiments performed in triplicate; ***, *P* < 0.001.

Next, we monitored in parallel the kinetics of apoptosis induction and cell cycle arrest, since PLK1 inhibition as well as vinca alkaloids are known to interfere with cell cycle progression. These experiments revealed that BI 2536 and VCR cooperated to cause a significant G2/M phase arrest as indicated by tetraploid DNA content (4n) already at 12 hours prior to the induction of DNA fragmentation, used as a typical marker of apoptosis (Figure 3A–3B, Supplementary Figure 2). Since flow cytometry-based analysis of cell cycle distribution cannot discriminate between M and G2 phase, we additionally assessed phosphorylation of histone H3 at serine 10 (pH3) as a specific marker of mitosis [16]. BI 2536/VCR co-treatment increased phosphorylation of histone H3 compared to treatment with either drug alone (Figure 3C), underscoring that BI 2536/VCR co-treatment arrests cells in mitosis.

To explore whether mitotic arrest is necessary for apoptosis induction we used the CDK1 inhibitor RO-3306 to prevent cells from entering mitosis. RO-3306 inhibited mitotic entry, as documented by lack of histone H3 phosphorylation upon addition of RO-3306 to BI 2536/VCR-treated cells (Figure 3D), and significantly reduced BI 2536/VCR-induced apoptosis (Figure 3E). The observed cytotoxicity of RO-3306 alone is consistent with a recent report showing that inhibition of CDK1 can induce cell death in NB cells [17]. These experiments demonstrate that mitotic arrest is required for BI 2536/VCR-induced apoptosis.

### BI 2536 and VCR cooperate to inactivate antiapoptotic BCL-2 proteins

**Figure 4:**
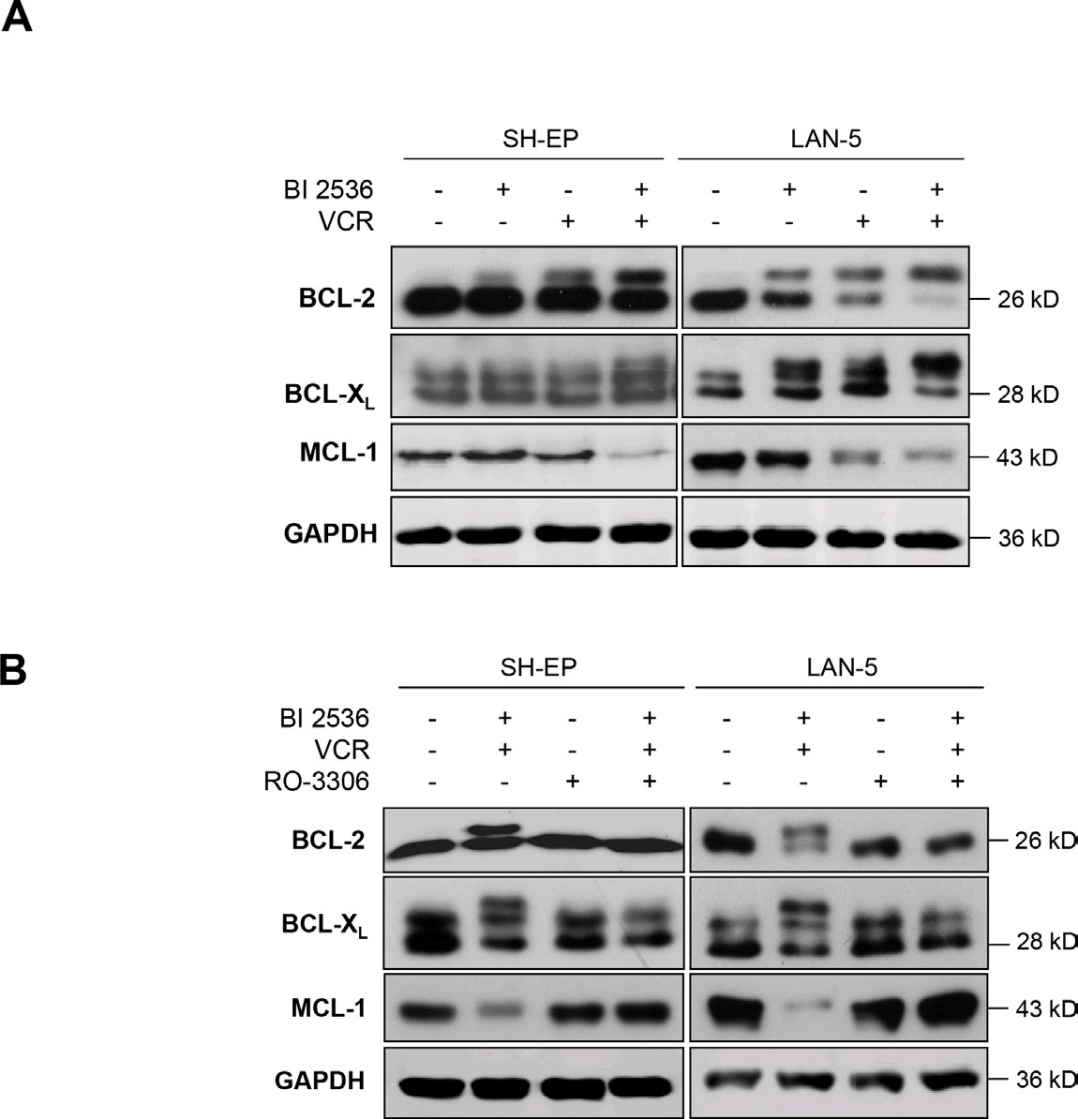
Mitotic arrest inactivates antiapoptotic BCL-2 proteins **A.** SH-EP cells were treated with 7.5 nM BI 2536 and/or 5 nM VCR, LAN-5 cells with 30 nM BI 2536 and/or 30 nM VCR for 24 hours. Expression of BCL-2, BCL-XL and MCL-1 was analyzed by Western blotting, expression of GAPDH served as loading control. Representative blots of three independent experiments are shown. **B.** SH-EP and LAN-5 cells were treated for 24 hours with BI 2536 and/or VCR as described in (A) in the presence or absence of 10 μM RO-3306. Expression of BCL-2, BCL-XL and MCL-1 was analyzed by Western blotting, expression of GAPDH served as loading control. Representative blots of two independent experiments are shown.

Since prolonged mitotic arrest has been described to cause changes in antiapoptotic BCL-2 family proteins [18, 19], we next monitored expression levels of BCL-2, BCL-X_L_ and MCL-1 by Western blot analysis. Indeed, BI 2536/VCR co-treatment altered the migration of BCL-2 and BCL-X_L_ on SDS-PAGE gel accompanied by upward band shifts that are consistent with increased phosphorylation (Figure 4A). In addition, BI 2536/VCR co-treatment caused decreased protein expression of MCL-1 (Figure 4A). To explore whether these changes in antiapoptotic BCL-2 proteins are linked to mitotic arrest, we added RO-3306 to the experiments in order to prevent mitotic arrest. Preventing cells from entering mitosis by addition of RO-3306 also abolished the BI 2536/VCR-stimulated phosphorylation of BCL-2 and BCL-X_L_ as well as MCL-1 downregulation (Figure 4B). This underscores the hypothesis that mitotic arrest is required for BI 2536/VCR-mediated inactivation of antiapoptotic BCL-2 proteins.

### BI 2536/VCR-mediated downregulation of MCL-1 during mitotic arrest contributes to apoptosis

**Figure 5:**
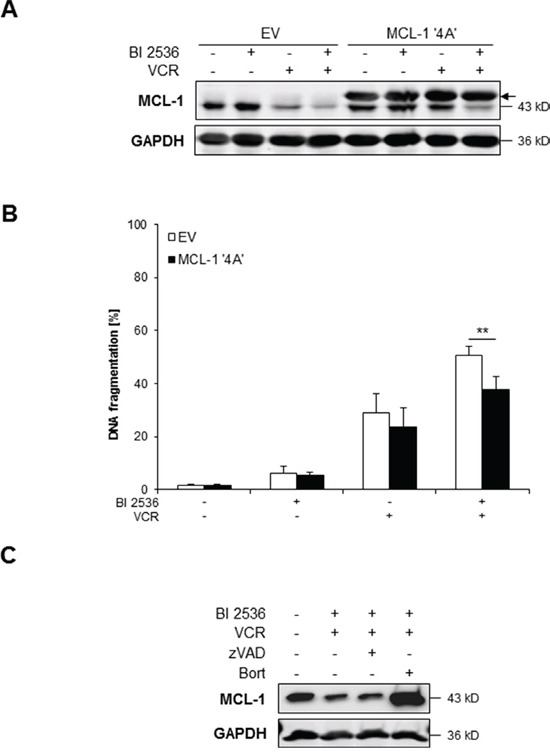
BI 2536/VCR-mediated downregulation of MCL-1 contributes to apoptosis **A–B.** SH-EP cells were transfected with non-degradable phospho-defective mutant of MCL-1 (MCL-1 ‘4A’) or empty vector (EV) and treated with 7.5 nM BI 2536 and/or 5 nM VCR for 24 hours (A) or 48 hours (B). Expression of MCL-1 was analyzed by Western blotting, expression of GAPDH served as loading control; arrow indicates exogenously expressed MCL-1 (A). Representative blots of two independent experiments are shown. Apoptosis was determined by analysis of DNA fragmentation of PI-stained nuclei using flow cytometry (B). Data are shown as mean +/− SD of three independent experiments performed in triplicate; **, *P* < 0.01. **C.** SH-EP cells were treated for 24 hours with 7.5 nM BI 2536 and/or 5 nM VCR, 20 μM zVAD.fmk and 50 nM Bortezomib. Expression of MCL-1 was analyzed by Western blotting, expression of GAPDH served as loading control. Representative blots of two independent experiments are shown.

Since MCL-1 has been reported to be phosphorylated during prolonged mitotic arrest leading to its proteasomal degradation [19], we asked whether phosphorylation-mediated degradation of MCL-1 is required for apoptosis induction. To address this question, we used a non-degradable phospho-defective mutant of MCL-1 (i.e. MCL-1 ‘4A’) that is mutated in four critical residues and thus resistant to phosphorylation and subsequent proteasomal degradation during mitosis [19]. Ectopic expression of MCL-1 ‘4A’ mutant significantly reduced BI 2536/VCR-mediated apoptosis (Figure 5A–5B). To confirm that downregulation of MCL-1 protein upon BI 2536/VCR co-treatment is due to proteasomal degradation rather than proteolytic cleavage, we tested the effects of the proteasomal inhibitor Bortezomib and the caspase inhibitor zVAD.fmk on BI 2536/VCR-stimulated decrease of MCL-1 protein. Bortezomib but not zVAD.fmk prevented MCL-1 downregulation upon BI 2536/VCR co-treatment (Figure 5C). This set of experiments shows that BI 2536/VCR-mediated proteasomal degradation of MCL-1 during mitotic arrest contributes to BI 2536/VCR-induced apoptosis.

### BCL-2 overexpression protects against BI 2536/VCR-induced apoptosis

**Figure 6:**
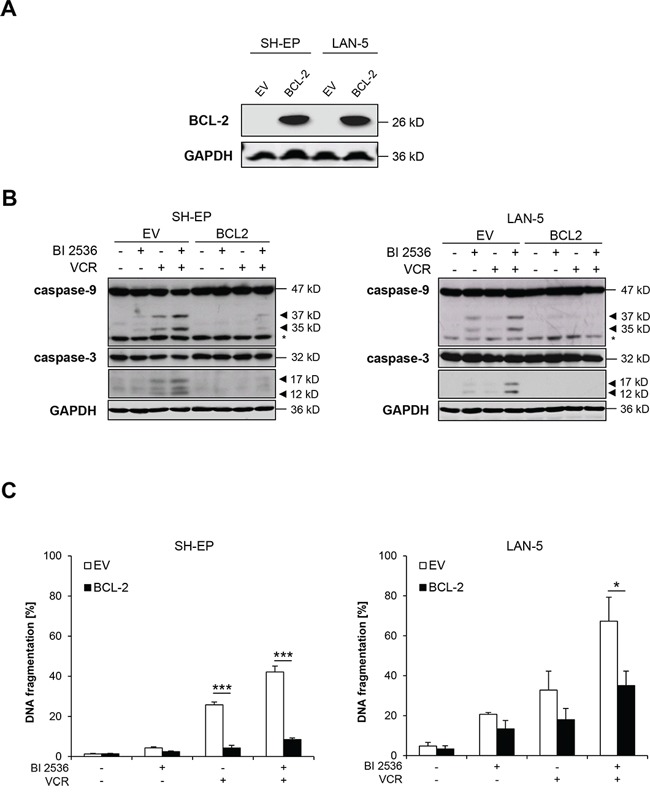
BCL-2 overexpression rescues BI 2536/VCR-induced apoptosis **A.** SH-EP and LAN-5 cells were stably transduced with empty vector or murine BCL-2 and BCL-2 expression was analyzed by Western blotting; expression of GAPDH served as loading control. **B.** SH-EP cells were treated with 7.5 nM BI 2536 and/or 5 nM VCR, LAN-5 cells with 30 nM BI 2536 and/or 30 nM VCR for 24 hours. Activation of caspase-9 and -3 was analyzed by Western blotting. Arrowheads indicate active cleavage fragments, expression of GAPDH served as loading control, asterisk denotes unspecified bands. Representative blots of two independent experiments are shown. **C.** SH-EP and LAN-5 cells were treated with BI 2536 and/or VCR for 48 hours as described in (B). Apoptosis was determined by analysis of DNA fragmentation of PI-stained nuclei using flow cytometry. Data are shown as mean +/− SD of three independent experiments performed in triplicate; *, *P* < 0.05; ***, *P* < 0.001.

Since BCL-2 has been described to become inactivated upon phosphorylation during prolonged mitotic arrest, we overexpressed BCL-2 (Figure 6A) to test the functional relevance of BCL-2 for BI 2536/VCR-induced apoptosis. Ectopic expression of BCL-2 inhibited BI 2536/VCR-triggered activation of caspase-9 and -3 (Figure 6B) and significantly reduced BI 2536/VCR-mediated apoptosis (Figure 6C). This indicates that phosphorylation-imposed inactivation of BCL-2 during mitotic arrest contributes to BI 2536/VCR-induced apoptosis.

### BI 2536 and VCR cooperate to activate BAX/BAK

**Figure 7:**
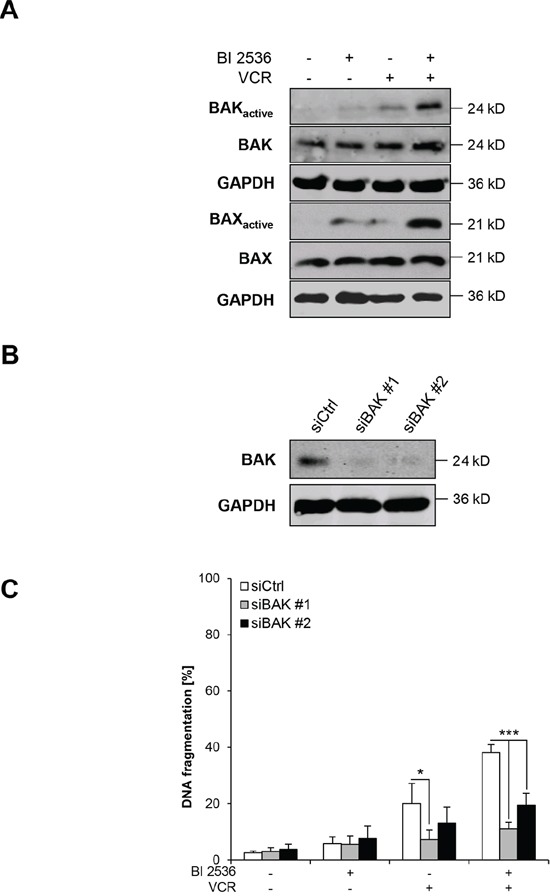
BI 2536/VCR-mediated activation of BAK and BAX is required for apoptosis **A.** SH-EP cells were treated with 7.5 nM BI 2536 and/or 5 nM VCR for 21 hours. BAK or BAX were immunoprecipitated using active conformation-specific antibodies and expression of active and total BAK or BAX was analyzed by Western blotting, GAPDH served as loading control. Representative blots of two independent experiments are shown. **B–C**: SH-EP cells were transiently transfected with non-silencing siRNA (siCtrl) or two different constructs targeting BAK (siBAK #1 and siBAK #2). Expression of BAK was analyzed by Western blotting, GAPDH served as loading control (B) SH-EP cells were treated with 7.5 nM BI 2536 and/or 5 nM VCR for 48 hours and apoptosis was determined by analysis of DNA fragmentation of PI-stained nuclei using flow cytometry (C) Data are shown as mean +/− SD of three independent experiments performed in triplicate; *, *P* < 0.05; ***, *P* < 0.001.

In light of our findings showing that BI 2536/VCR co-treatment leads to inactivation of antiapoptotic BCL-2 proteins, we next asked whether this in turn results in activation of the proapoptotic proteins BAX and BAK. To address this question, we performed immunoprecipitation using active conformation-specific antibodies to determine the activation status of BAX and BAK by conformational changes. BI 2536 and VCR cooperated to trigger activation BAX and BAK (Figure 7A). To test the functional relevance of this finding, we knocked down BAK by RNA interference (RNAi) (Figure 7B). Silencing of BAK using two distinct siRNA oligonucleotides significantly rescued BI 2536/VCR-induced apoptosis (Figure 7C). These experiments show that BI 2536 and VCR cooperate to trigger BAX/BAK activation, which is required for BI 2536/VCR-induced apoptosis.

### BI 2536/VCR co-treatment suppresses NB tumor growth *in vivo*

**Figure 8:**
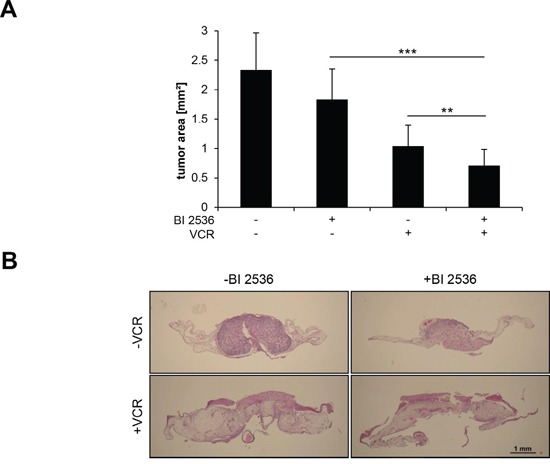
BI 2536 and VCR cooperate to suppress tumor growth *in vivo* SH-EP cells were implanted on the CAM of fertilized chicken eggs at day 8 of incubation and treated with 7.5 nM BI 2536 and/or 5 nM VCR for three consecutive days. Tumors were dissected with surrounding CAM and tumor area was analyzed in hematoxylin and eosin (HE)-stained sections. Data are shown as mean + SD (**A.** *n* = 15–19 eggs) or as representative images of HE-stained sections (**B.** scale bar: 1 mm). **, *P* < 0.01; ***, *P* < 0.001.

Finally, we investigated the antitumor activity of BI 2536/VCR co-treatment against NB *in vivo* using the chorioallantoic membrane (CAM) model, an *in vivo* tumor model for anticancer drug testing [20, 21]. NB cells were seeded on the CAM of chicken embryos, allowed to form tumors and treated with BI 2536 and/or VCR for three days. BI 2536/VCR co-treatment significantly reduced tumor growth compared to single treatment (Figure 8). This demonstrates that BI 2536/VCR co-treatment cooperates to suppress tumor growth in a NB model *in vivo*.

## DISCUSSION

Searching for rational combination treatments, we investigated the potential of the PLK1 inhibitor BI 2536 to sensitize NB cells for chemotherapy. In the present study, we discover that BI 2536 synergizes with vinca alkaloids to trigger apoptosis in NB cells. Moreover, BI 2536 acts together with VCR to suppress long-term clonogenic survival and to inhibit tumor growth in an *in vivo* model of NB.

Mechanistic studies reveal that BI 2536 and VCR act in concert to cause mitotic arrest that results in phosphorylation-mediated inactivation of BCL-2 and BCL-X_L_ as well as downregulation of MCL-1. Inactivation of antiapoptotic BCL-2 proteins in turn promotes BAX and BAK activation, leading to cleavage of caspase-9 and -3 and caspase-dependent apoptosis. Several lines of genetic evidence support our conclusion that differential modulation of BCL-2 family proteins in favor of proapoptotic proteins during mitotic arrest is required for BI 2536/VCR-induced apoptosis, since i) ectopic expression of a MCL-1 phospho-mutant, ii) BCL-2 overexpression or iii) BAK knockdown significantly decrease BI 2536/VCR-mediated apoptosis. Together, these data demonstrate that the engagement of the mitochondrial pathway of apoptosis is necessary for BI 2536/VCR-induced apoptosis. Prolonged mitotic arrest has previously been reported to disable antiapoptotic BCL-2 proteins via phosphorylation, leading to their inactivation (i.e. BCL-2 and BCL-X_L_) or proteasomal degradation (i.e. MCL-1) [18, 19, 22, 23]. In line with this notion, phospho-defective mutants of BCL-2, BCL-X_L_ or MCL-1 have been demonstrated to inhibit cell death during mitosis [18, 19, 22–25].

Our study has several important implications. Foremost, it emphasizes that combination regimens with small-molecule PLK1 inhibitors and vinca alkaloids warrant further evaluation in NB. The PLK1 inhibitor BI 6727 as monotherapy has recently been shown in an evaluation by the Pediatric Preclinical Testing Program to exhibit intermediate or high activity in NB xenografts [14]. Also, Ackermann et al. reported significant suppression of tumor formation and of established tumor growth by treatment with BI 2536 in NB xenograft models [8]. However, Gorlick et al. also raised some concerns that exposure to BI 6727 in rodents that lead to regressions may be greater than can be achieved in humans [14]. In addition, toxicity limited the evaluation of BI 6727 in the acute lymphoblastic leukemia *in vivo* panel [14]. Drug combinations can offer advantages, as relatively low concentrations of each drug may achieve synergistic antitumor activity without causing additive toxicity. This underscores the relevance of our key finding showing synergism of PLK1 inhibitor-based combination therapies together with vinca alkaloids in preclinical *in vitro* and *in vivo* models of NB. We recently reported that systemic administration of BI 6727 and VCR cooperated to significantly suppress tumor growth *in vivo* without causing additive toxicity using a human xenograft mouse model of rhabdomyosarcoma [26], pointing to some tumor selectivity of this combination treatment.

The feasibility of transferring this approach into future experimental treatment protocols is underscored by the fact that vinca alkaloids such as VCR are part of standard therapy for NB and that the PLK1 inhibitor BI 6727 as single agent is under evaluation in a phase I clinical trial in pediatric malignancies (http://www.clinicaltrials.gov). Moreover, previous studies highlighted the significance of PLK1 as a therapeutic target in NB. High expression levels of PLK1 have been detected in high-risk NB and were associated with several unfavorable prognostic parameters [8]. Using a small-molecule kinase inhibitor screen, PLK1 was identified as a critical regulator of survival and self-renewal in NB tumor-initiating cells that have been implicated in sustaining tumor growth, progression and metastasis [27]. Taken together, PLK1 inhibitors represent promising compounds to sensitize NB for chemotherapy that warrant further investigation.

## MATERIALS AND METHODS

### Cell culture and chemicals

NB cell lines with different MYCN status (SH-EP: MYCN non-amplified; LAN-5: MYCN amplified; NLF: MYCN amplified) were obtained from American Type Culture Collection (ATCC) (Manassas, VA, USA) and maintained in DMEM GlutaMAX™-l or RPMI medium (Life Technologies, Inc., Darmstadt, Germany), supplemented with 10% fetal calf serum (FCS), 1% penicillin/streptomycin, 1 mM sodium pyruvate and 10 mM HEPES (all from Life Technologies, Inc.). LAN-5 cells were grown in flasks coated with collagen (BD Biosciences, San Jose, CA, USA). VCR, VBL and VNR were purchased from Sigma (Deisenhofen, Germany); RO-3306 from Merck (Darmstadt, Germany); zVAD.fmk from Bachem (Heidelberg, Germany). MCL-1 plasmids (pCMV-Tag 3B, MCL-1 ‘4A’) were kindly provided by Genentech, Inc. (South San Francisco, CA, USA). Chemicals were purchased from Sigma unless otherwise indicated.

### Determination of apoptosis, cell density and colony formation

Apoptosis was determined by flow cytometric analysis (FACSCanto II, BD Biosciences, Heidelberg, Germany) of DNA fragmentation of propidium iodide (PI)-stained nuclei as described previously [28]. Cell density was assessed by crystal violet assay using crystal violet solution (0.5% crystal violet, 30% ethanol, 3% formaldehyde). Plates were then rinsed with water and crystal violet incorporated by the cells was re-solubilized in a solution containing 1% SDS. Absorbance at 550 nm was measured using a microplate reader (Infinite M100, Tecan, Crailsheim, Germany). Results are expressed as percentage of cell density relative to untreated controls. For colony formation assay, 100 cells per well were seeded in 6-well plates and treated for 12 hours with indicated drug concentrations. Cells were cultured in drug-free medium for additional 13 days before fixation and staining with 0.5% crystal violet, 30% ethanol and 3% formaldehyde. Colonies were counted macroscopically.

### Overexpression and RNA interference

Stable overexpression of murine BCL-2 was performed by lentiviral vectors. Shortly, Phoenix cells were transfected with 20 μg of pMSCV plasmid (empty vector; BCL-2) using calcium phosphate transfection. Virus-containing supernatant was collected, sterile-filtered, and used for spin transduction at 37°C in the presence of 8 μg/ml polybrene. For selection, 2 μg/ml (SH-EP) or 20 μg/ml (LAN-5) blasticidin (Invitrogen) were used. For transient overexpression, SH-EP cells were transfected with 4 μg of pCMV-Tag 3B plasmid (empty vector; MCL-1 ‘4A’ (S64A/S121A/S159A/T163A)), supplied with Lipofectamine 2000 (Life Technologies, Inc.) and selected with 500 μg/ml G418 (Carl Roth, Karlsruhe, Germany). For transient knockdown of BAK, cells were reversely transfected with 5 pmol Silencer Select (Life Technologies, Inc.) control siRNA (4390844) or targeting siRNA (s1880 and s1881 for BAK), respectively, using Lipofectamine RNAiMAX reagent (Life Technologies, Inc.) and Opti-MEM medium (Life Technologies, Inc.).

### Determination of BAK and BAX activation

BAK and BAX activation was determined by immunoprecipitation as previously described [21]. Briefly, cells were lysed in CHAPS lysis buffer (10 mmol/l HEPES, pH 7.4; 150 mmol/l NaCl; 1% CHAPS). 500 μg protein was incubated over night at 4°C with 0.5 μg mouse anti-BAK antibody (AB-1; Calbiochem) or 8 μg mouse anti-BAX antibody (clone 6A7; Sigma) and 10 μl pan-mouse IgG Dynabeads, washed with CHAPS lysis buffer, and analyzed by Western blotting using rabbit anti-BAK antibody or rabbit anti-BAX NT antibody.

### Cell cycle analysis

Cells were stained with PI as described previously [28]. Cell cycle analysis was performed using FlowJo Software (Tree Star Inc, Oregon, USA) following manufacturer's instructions.

### CAM assay

CAM assay was done as described previously [29]. Briefly, 1 × 10^6^ SH-EP cells were implanted on fertilized chicken eggs on day 8 of incubation and were treated with 34.31 ng BI 2536 and/or 40.48 ng VCR (corresponding to 7.5 nM BI 2536 and 5 nM VCR in cell culture experiments) for three consecutive days, sampled with the surrounding CAM, fixed in 4% paraformaldehyde, embedded in paraffin, cut in 3 μm sections and then analyzed by immunohistochemistry using 1:1 hematoxylin and 0.5% eosin. Images were digitally recorded and tumor areas were analyzed with ImageJ digital imaging software (NIH, Bethesda, MD, USA).

### Western blot analysis

Western blot analysis was performed as described previously [28] using the following antibodies: BAK, BCL-2, BCL-X_L_ (BD, New Jersey, USA), BCL-2 (Life Technologies, Inc.), caspase-3, caspase-9 (Cell Signaling, Beverly, MA), BAX NT, pH3 (Millipore, Darmstadt, Germany), MCL-1 (Enzo Life Science, Lörrach, Germany), GAPDH (HyTest, Turku, Finland). Goat anti-mouse IgG and goat anti-rabbit IgG conjugated to horseradish peroxidase (Santa Cruz Biotechnology) as secondary antibodies and enhanced chemiluminescence (Amersham Bioscience, Freiburg, Germany) or infrared dye-labeled secondary antibodies and infrared imaging were used for detection (Odyssey Imaging System, LI-COR Bioscience, Bad Homburg, Germany). Representative blots of at least two independent experiments are shown.

### Statistical analysis

Statistical significance was assessed by Student's *t*-Test (two-tailed distribution, two-sample, equal variance). Drug interactions were analyzed by the CI method based on that described by Chou [30] using CalcuSyn software (Biosoft, Cambridge, UK). CI < 0.9 indicates synergism, 0.9–1.1 additivity and > 1.1 antagonism.

## SUPPLEMENTARY TABLE AND FIGURES




