# Supplementary material for: Polo-like kinase 1 inhibition sensitizes neuroblastoma cells for vinca alkaloid-induced apoptosis
Source: Oncotarget. 2015 May 14;7(8):8700–11. doi: 10.18632/oncotarget.3901 (PMC4890998; doi:10.18632/oncotarget.3901)
Supplement: Supplementary file 1 [file oncotarget-07-8700-s001.pdf]

SUPPLEMENTARY TABLE AND FIGURES

Supplementary Table 1: Synergistic induction of apoptosis by BI 2536 and vinca alkaloids. Combination indices (CI) were calculated as described in materials and methods for apoptosis induced by combined treatment for 48 hours with indicated concentrations of BI 2536 and vinca alkaloids. Synergistic drug combinations are indicated in bold letters.

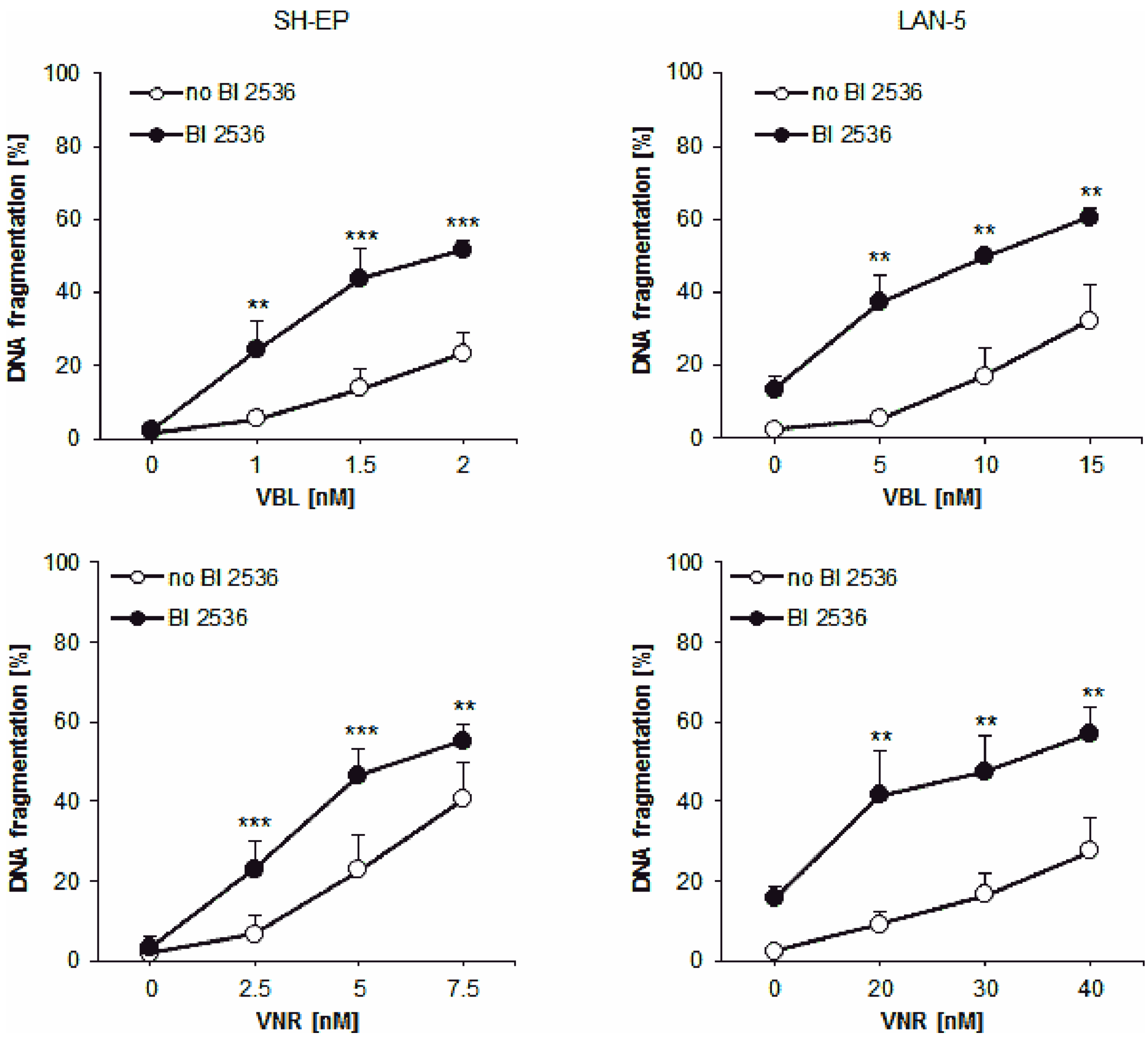

Supplementary Figure 1: BI 2536 synergizes with microtubule-destabilizing drugs to induce cell death in NB cells. SH-EP cells were treated 48 hours with 5 nM BI 2536 and/or indicated concentrations of VBL and VNR, LAN-5 cells with 30 nM BI 2536 and/or indicated concentrations of VBL and VNR. Apoptosis was determined by analysis of DNA fragmentation of PI-stained nuclei using flow cytometry. Data are shown as mean +/− SD of three independent experiments performed in triplicate. *, $P < 0.05$; **, $P < 0.01$; ***, $P < 0.001$.

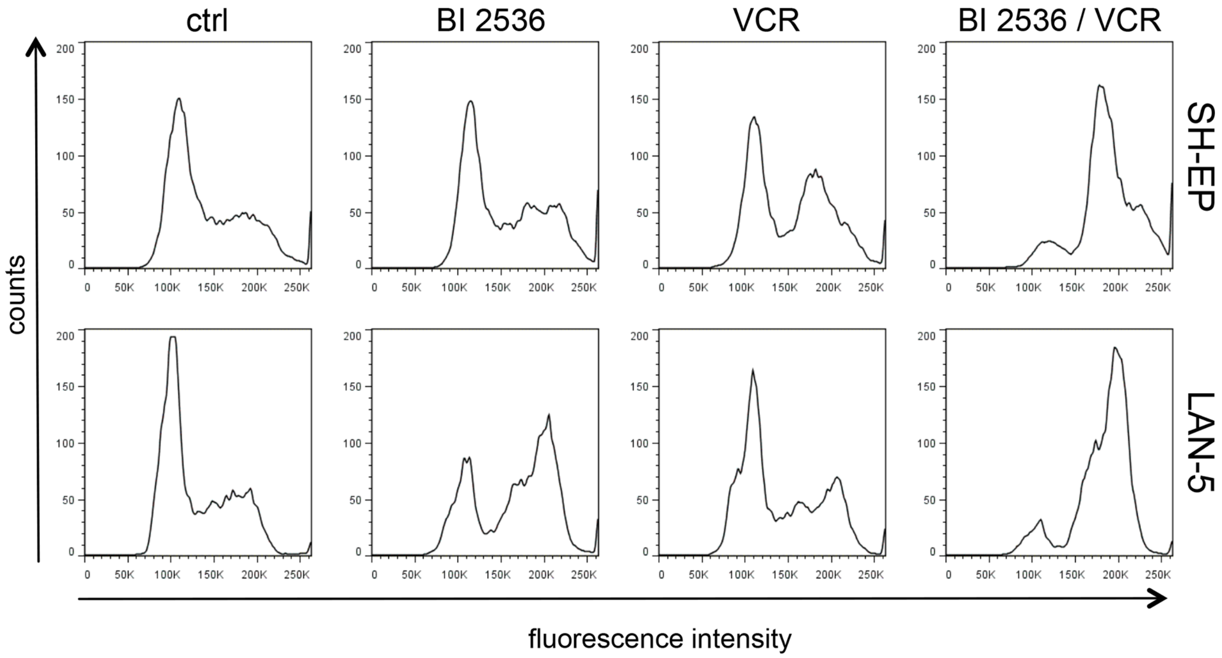

Supplementary Figure 2: Mitotic arrest is required for BI 2536/VCR-induced apoptosis. For cell cycle analysis, SH-EP cells were treated for 12 hours with 7.5 nM BI 2536 and/or 10 nM VCR, LAN-5 cells with 30 nM BI 2536 and/or 75 nM VCR. DNA was stained with PI and cell cycle analysis was performed using FlowJo software. Representative histograms for control and treated samples are shown.
