# Supplementary material for: Polo-like kinase 1 inhibition sensitizes neuroblastoma cells for vinca alkaloid-induced apoptosis
Source: Oncotarget. 2015 May 14;7(8):8700–11. doi: 10.18632/oncotarget.3901 (PMC4890998; doi:10.18632/oncotarget.3901)
Supplement: Supplementary file 2 [file oncotarget-07-8700-s002.pptx]

## Slide 1
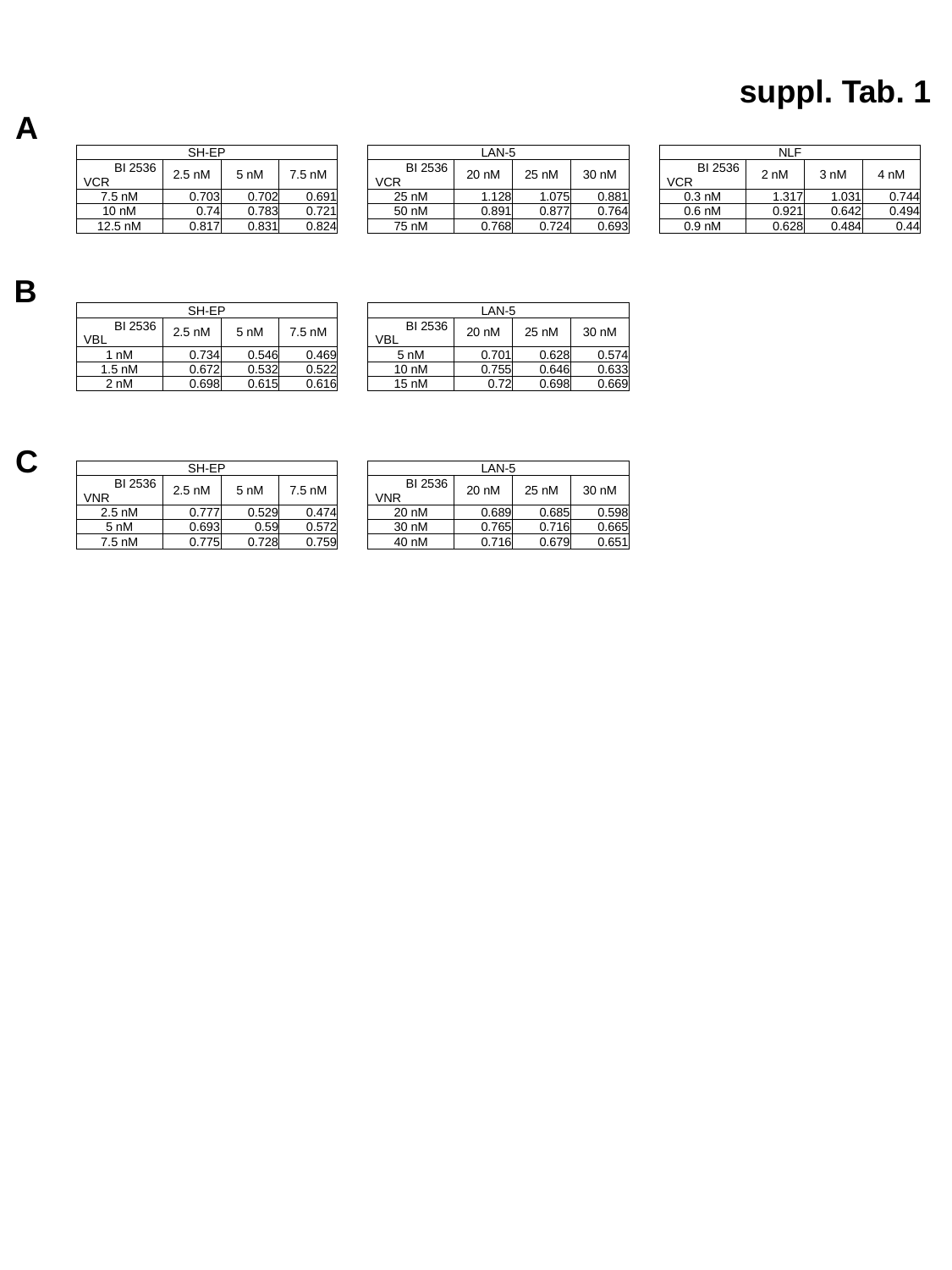

suppl. Tab. 1
A
| SH-EP | | | | | LAN-5 | | | | | NLF | | | |
| --- | --- | --- | --- | --- | --- | --- | --- | --- | --- | --- | --- | --- | --- |
| BI 2536 VCR | 2.5 nM | 5 nM | 7.5 nM | | BI 2536 VCR | 20 nM | 25 nM | 30 nM | | BI 2536 VCR | 2 nM | 3 nM | 4 nM |
| 7.5 nM | 0.703 | 0.702 | 0.691 | | 25 nM | 1.128 | 1.075 | 0.881 | | 0.3 nM | 1.317 | 1.031 | 0.744 |
| 10 nM | 0.74 | 0.783 | 0.721 | | 50 nM | 0.891 | 0.877 | 0.764 | | 0.6 nM | 0.921 | 0.642 | 0.494 |
| 12.5 nM | 0.817 | 0.831 | 0.824 | | 75 nM | 0.768 | 0.724 | 0.693 | | 0.9 nM | 0.628 | 0.484 | 0.44 |
| | | | | | | | | | | | | | |
| SH-EP | | | | | LAN-5 | | | | | | | | |
| BI 2536 VBL | 2.5 nM | 5 nM | 7.5 nM | | BI 2536 VBL | 20 nM | 25 nM | 30 nM | | | | | |
| 1 nM | 0.734 | 0.546 | 0.469 | | 5 nM | 0.701 | 0.628 | 0.574 | | | | | |
| 1.5 nM | 0.672 | 0.532 | 0.522 | | 10 nM | 0.755 | 0.646 | 0.633 | | | | | |
| 2 nM | 0.698 | 0.615 | 0.616 | | 15 nM | 0.72 | 0.698 | 0.669 | | | | | |
| | | | | | | | | | | | | | |
| SH-EP | | | | | LAN-5 | | | | | | | | |
| BI 2536 VNR | 2.5 nM | 5 nM | 7.5 nM | | BI 2536 VNR | 20 nM | 25 nM | 30 nM | | | | | |
| 2.5 nM | 0.777 | 0.529 | 0.474 | | 20 nM | 0.689 | 0.685 | 0.598 | | | | | |
| 5 nM | 0.693 | 0.59 | 0.572 | | 30 nM | 0.765 | 0.716 | 0.665 | | | | | |
| 7.5 nM | 0.775 | 0.728 | 0.759 | | 40 nM | 0.716 | 0.679 | 0.651 | | | | | |
B
C
